# Levan from *Leuconostoc citreum* BD1707: production optimization and changes in molecular weight distribution during cultivation

**DOI:** 10.1186/s12896-021-00673-y

**Published:** 2021-02-04

**Authors:** Jin Han, Huafeng Feng, Xiaohua Wang, Zhenmin Liu, Zhengjun Wu

**Affiliations:** State Key Laboratory of Dairy Biotechnology, Shanghai Engineering Center of Dairy Biotechnology, Research Institute of Bright Dairy & Food Co., Ltd., Shanghai, 200436 China

**Keywords:** *Leuconostoc citreum* BD1707, Levan, Optimization, Distribution of molecular weight, Levansucrase

## Abstract

**Background:**

Levan is a well-known homopolymer of fructose composed predominantly of β-(2, 6) fructofuranosyl linkages in the backbone with occasional β-(2, 1) linkages in the branch chains with varied applications. However, high production cost due to low yield of microbial levan has become a bottleneck for its practical applications. Furthermore, factors affecting the molecular mass of the synthesized levan by *Leuconostoc* spp. during prolonged cultivation is not fully elucidated.

**Methods:**

The cultivation condition for *Leuconostoc citreum* BD1707 to synthesize levan was optimized by single-factor experiments and subsequently with response surface methodology (RSM). The average molecular weight (Mw) of levan synthesized by the strain *L.citreum* BD1707 under the optimized cultivation conditions was monitored by high-performance size exclusion chromatography (HPSEC). Finally, the enzyme with levan-degrading activity was determined by sodium dodecyl sulfate polyacrylamide gel electrophoresis (SDS-PAGE).

**Results:**

The levan yield of BD1707 reached 34.86 g/L with a corresponding productivity of 7.47 g/L/d under the optimal cultivation conditions deduced by RSM, i.e., cultivation at 26 °C and 200 rpm for 112 h in tomato juice supplemented with 172 g/L sucrose with an initial pH value of 6.12. The Mw of levan reached a peak value of 2.320 × 10^7^ Da at 6 h of cultivation under the optimized cultivation conditions and then gradually decreased to 8.809 × 10^6^ Da after 120 h of cultivation.

**Conclusion:**

The levan yield of the strain *L.citreum* BD1707 could be sufficiently enhanced via cultivation condition optimization. The decrease in molecular mass of the synthesized levan was attributed predominantly to the hydrolytic activity of levansucrase secreted by *L.citreum* BD1707 during cultivation, with an estimated Mw of 130 KD by SDS-PAGE, while the effect of acid hydrolysis could be nearly neglected.

**Supplementary Information:**

The online version contains supplementary material available at 10.1186/s12896-021-00673-y.

## Background

Levan is a well-known homopolymer of fructose composed predominantly of β-(2, 6) fructofuranosyl linkages in the backbone with occasional β-(2, 1) linkages in the branch chains and is widespread in both of plants and microorganisms. Levan from different sources differs in molecular weight and degree of branching. The molecular weight of bacterial levan (ranging from 2.0 × 10^6^ to 1.0 × 10^8^ Da) is generally much higher than that of levan from plants (ranging from 2000 to 3,733,000 Da) [1]. Microbial levan is synthesized by the transglycosylation activity of levansucrase (sucrose: 2–6-β-D-fructan 6-β-D-fructosyltransferase, E.C.2.4.1.10) in the presence of sucrose. Levansucrase, belonging to the glycoside hydrolase family 68 (GH68) [2] has been reported in a variety of microorganisms, such as *Bacillus* and *Pseudomonas* species and a few lactic acid bacteria (LAB) [3]. Due to its well water solubility, high molecular mass and low viscosity, microbial levan can be used as emulsifing, stabilizing, thickening and encapsulating agent in the food industry [3] and as blood plasma volume extender [4], antiobesity agent or hypocholesterolemic agent, etc., in the pharmaceutical industry [5]. Furthermore, owing to its moisturizing properties, low cell cytotoxicity, promoting mammalian cell proliferation and anti-inflammatory effect, levan can also be utilized in cosmetic products [6].

However, low yield of microbial levan has become a bottleneck for its practical application [7]. In recent years, researchers have exploited various agricultural raw materials such as molasses [8, 9] and syrup [10, 11] to replace chemically defined medium (CDM) to reduce the production costs of levan.

The weight-average molecular weight (Mw) of microbial levan, an important index rendering this biopolymer with varied physiochemical and functional properties, is affected by the type of producer [12] and cultivation conditions, such as sucrose concentration [13], temperature [14], ionic strength [15] and initial pH value of the medium [16]. Calazans GTM et al. found that levan with Mw of 456,900 Da showed a superior antitumor activity than those with lower or higher molecular mass (Mw = 353,500 Da, 720,200 Da, 769,500 Da and 1,073,500 Da) and concluded that antitumor activity of levan was restricted to the biopolymer with molecular weight in specific ranges [17]. Because the Mw of levan might greatly affect its application, factors influencing the molecular mass of levan synthesized by individual microbial producer should be carefully checked.

Tomato (*Lycopersicon esculentum*) is one of the most widely cultivated vegetables of the family *Solanaceae*. Tomato juice is not only well recognized as a healthy beverage but also as a natural medium suitable for the growth and enrichment of beneficial metabolites of lactic acid bacteria (LAB). Tomato juice fermented by four LAB species (*Lactobacillus acidophilus*, *L. plantarum*, *L. casei* and *L. delbrueckii*) was suggested to be a probiotic beverage suitable for vegetarians as well as subjects allergic to dairy-based products [18].

In our previous study, *Leuconostoc citreum* BD1707 (=CGMCC 6431) was reported to synthesize levan in sucrose-supplemented tomato juice (tomato juice-sucrose medium, TJSM) with a yield of 28 g/L [19]. However, no study regarding the optimization of cultivation conditions on levan production as well as its molecular weight distribution by *Leuconostoc spp.* in tomato juice has been reported.

In the present study, the effects of cultivation period (h), temperature (°C), initial pH value of the medium and sucrose concentration (g/L) in TJSM on levan production by *L. citreum* BD1707 were investigated by single-factor experiments and response surface methodology (RSM). Furthermore, the growth characteristics of *L. citreum* BD1707 under optimized cultivation conditions, including changes in the levels of sucrose, fructose, glucose and levan production, were also assayed. Finally, the variation of the average molecular weight (Mw) of the levan synthesized in the broth was determined, which was gradually decreased during the prolonged cultivation of the strain *L*.*citreum* BD1707 and caused predominantly by enzyme(s) secreted by the producer cells with levan degrading activity.

## Materials and methods

### Bacterial strain, propagation and storage

*L. citreum* BD1707 (=CGMCC 6431) was provided by the State Key Laboratory of Dairy Biotechnology, Bright Dairy & Food Co., Ltd., Shanghai, China. The bacterial strain was routinely streaked on M17 agar (Merck, Germany) supplemented with 50 g/L sucrose and incubated at 30 °C aerobically. The strain was stored in M17 broth (Merck, Germany) supplemented with 10% glycerol at − 80 °C.

### Preparation of TJSM

TJSM was prepared according to the method described previously [20]. Briefly, tomatoes (*Solanum lycopersicum*) purchased from local market were cut into small cubes, ground in a pulper and filtered through cotton gauze to remove the majority of the peel and seeds. The filtrated tomato juice was further centrifuged to remove the fruit debris. The major biochemical components of the tomato juice were determined in triplicate and listed in Table 1. No obvious difference in the components was determined among different variety of tomato (see Supplementary material, Figure S1, Table S1). The tomato juice was supplemented with sucrose at 50, 100, 150, 200 or 250 g/L, and the pH value of the mixture was adjusted to 4.5, 5.5, 6.5, 7.5, or 8.5 individually by addition of 5.0 M NaOH. The TJSM was sterilized at 121 °C for 20 min.

**Table 1:** Major biochemical components and parameters of tomato juice (values are the average ± range of triplicate (3) analyses)

Component/parameter	Level
Density	1.018 ± 0.002 g/100 mL
Ash	0.42 ± 0.01 g/100 mL
Free reducing sugars^a^	2.67 ± 0.03 g/100 mL
Total Kjeldahl nitrogen^b^	0.074 ± 0.001 g/100 mL
Fat^c^	0.10 ± 0.01 g/100 mL
pH^d^	4.15 ± 0.03

^a^ Determined by the DNS method^[52]^, using glucose as the standard

^b^ Determined using a Tecator Kjeltec 1025 system

^c^ Determined using a Tecator Soxtec HT2 system

^d^Determined using a Sartorius PB − 10 pH meter

### Single-factor experiment

To quantify the relationship between individual factor and the response variable (levan yield), single-factor experiments were conducted. In the present study, four factors, i.e., cultivation period, cultivation temperature, initial pH and sucrose concentration in TJSM, were investigated; these factors were presumed to significantly affect levan synthesis by the strain *L*.*citreum* BD1707.

A loop of fresh culture of the strain *L*.*citreum* BD1707 grown on M17 agar was inoculated into 100-mL Erlenmeyer flask containing 25 mL of sterile M17 broth and cultivated at 30 °C on a rotary shaker (Model I2400, New Brunswick Scientific Inc., Edison, NJ, U.S.) at 200 rpm for 24 h. The cells were collected by centrifugation at 15,000×g for 5 min at 4 °C and washed twice with sterile buffered physical saline. Subsequently, the washed cells were suspended in initial volume of buffered physical saline (9.2 log10 cfu/mL) and inoculated at a 2% (v/v) ratio into 250-mL Erlenmeyer flasks containing 100 mL of sterilized TJSM supplemented with varying levels of sucrose (50, 100, 150, 200 and 250 g/L) at different initial pH values (4.5, 5.5, 6.5, 7.5 or 8.5). The inoculated TJSM was then incubated at 15, 20, 25, 30 or 35 °C on a shaker at 200 rpm. Samples were withdrawn at 0, 24, 48, 72, 96 and 120 h to assay to the yield of levan as described below.

### RSM experimental design

RSM based on a Box-Behnken design (BBD), which was generated using Design Expert 8.0.5b software (Stat-Ease Corporation, USA), was utilized to investigate the interactions among the individual factors tested in the aforementioned single-factor experiment on levan production by BD1707. The variables and their coded levels were listed in Table 2. The three-level four-factor factorial BBD developed a total of 27 runs containing 3 replications of the central points (to check if there was a nonlinear relationship between the variables and the responses) and 24 trials organized in a fractional factorial design (Table 3). The experimental data were analyzed by the response surface regression procedure to fit the following second-order polynomial equation:

1 $$ \mathrm{Y}={\upbeta}_0+{\upbeta}_1\mathrm{A}+{\upbeta}_2\mathrm{B}+{\upbeta}_3\mathrm{C}+{\upbeta}_4\mathrm{D}+{\upbeta}_{11}{\mathrm{A}}^2+{\upbeta}_{22}{\mathrm{B}}^2+{\upbeta}_{33}{\mathrm{C}}^2+{\upbeta}_{44}{\mathrm{D}}^2+{\upbeta}_{12}\mathrm{AB}+{\upbeta}_{13}\mathrm{AC}+{\upbeta}_{14}\mathrm{AD}+{\upbeta}_{23}\mathrm{BC}+{\upbeta}_{24}\mathrm{BD}+{\upbeta}_{34}\mathrm{CD} $$

where Y was the predicted response corresponding to levan production; A, B, C and D were the coded independent variables; β_0_ is an offset term; β_1,_ β_2,_ β_3,_ and β_4_ are linear effects; β_11,_ β_22,_ β_33,_ and β_44_ are quadratic coefficients; and β_12,_ β_13,_ β_14,_ β_23,_ β_24,_ and β_34_ are interaction terms.

**Table 2:** Independent variables and their coded levels chosen for BBD

Variable code	Variable	Level		
		−1	0	+ 1
A	Cultivation time (h)	72	96	120
B	Cultivation temperature (°C)	20	25	30
C	Initial pH	5.5	6.5	7.5
D	Sucrose concentration (g/L)	100	150	200

**Table 3:** Box-Behnken design (matrix and responses) for levan production by BD1707

Run	A	B	C	D	Levan production (g/L)	
					Experimental	Predicted
1	−1	0	1	0	31.72	31.82
2	0	0	0	0	33.40	33.36
3	0	0	−1	−1	21.92	22.47
4	1	0	−1	0	33.16	32.87
5	0	−1	0	−1	11.46	10.30
6	1	1	0	0	27.88	28.47
7	0	0	0	0	32.94	33.36
8	0	0	1	1	30.97	30.62
9	−1	0	−1	0	27.28	28.42
10	0	1	0	−1	19.94	19.84
11	0	1	−1	0	28.77	27.76
12	1	0	1	0	31.44	30.10
13	0	1	1	0	27.95	27.50
14	−1	−1	0	0	17.60	17.21
15	−1	0	0	1	29.96	29.69
16	0	−1	−1	0	16.84	17.29
17	−1	1	0	0	27.36	27.37
18	1	0	0	1	31.80	32.38
19	1	−1	0	0	18.64	18.84
20	0	1	0	1	27.08	28.04
21	0	0	1	−1	22.30	23.34
22	1	0	0	−1	22.96	23.22
23	−1	0	0	-1	23.76	23.17
24	0	-1	1	0	17.18	18.18
25	0	-1	0	1	17.88	17.79
26	0	0	0	0	33.74	33.36
27	0	0	-1	1	31.71	30.87

### Preparation and quantification of Levan

Levan in the cultivated broth was prepared according to the procedure described previously [19]. In brief, the cultivated broth was centrifuged at 15,000×g for 5 min at 4 °C, and 4 volumes of prechilled ethanol were added to the supernatant. The mixture was stored at 4 °C overnight. The precipitate was collected by centrifugation at 15,000×g at 4 °C for 20 min and washed twice with prechilled ethanol. The pellet was redissolved in deionized water, dialyzed (MWCO 14,000 Da) against deionized water at 4 °C for 72 h, freeze-dried using FreeZone12 (Labconco corporation, Kansas, USA) and weighed. As reported previously, the lyophilized powder was composed solely of levan [19].

### Change of the molecular weights of Levan during prolonged bacterial cultivation

The change of the molecular weights of levan in the cultivated TJSM under optimized conditions, except for the cultivation period, was investigated. The resuspended BD1707 cells were inoculated at a 2% (v/v) ratio into 250-mL Erlenmeyer flasks containing 100 mL of sterile TJSM supplemented with 172 g/L sucrose, with the initial pH value adjusted to 6.12. The inoculated broth was cultivated at 26 °C on a shaker at 200 rpm. Samples at 3, 6, 12, 24 and 120 h of cultivation were withdrawn and treated as mentioned above to prepare levan for further Mw assays.

### Influence of Levan-degrading enzymes and organic acids on the mw of Levan

The suspended BD1707 cells were inoculated at a 2% (v/v) ratio into 250-mL Erlenmeyer flasks containing 100 mL of sterile TJSM supplemented with 172 g/L sucrose, with the initial pH value adjusted to 6.12.

The inoculated broth was first cultivated at 26 °C on a shaker at 200 rpm for 72 h. Then, the cultivated broth was divided into 4 groups undertaking different treatments: (A) the cultivated broth was centrifuged, precipitated by absolute alcohol, dialyzed against deionized water and lyophilized to prepare levan as aforementioned; (B) the cultivated broth was centrifuged at 15,000×g for 5 min at 4 °C to remove bacterial cells, and the supernatant (with a pH value of 4.00) was filtrating sterilized with a 0.22-μm membrane (Sartorius AG, Goettingen, GER); (C) The pH value of the cell free supernatant as obtained in group B was firstly neutralized to 7.0 with 1 M NaOH and then boiled for 5 min to inactivate enzymes capable of degrading levan. Then, the pH of the heat treated supernatant was readjusted to 4.00 (the predicted pH of 120-h fermented medium) after cooling to room temperature; (D) the control group without any treatment.

Group B to D were further incubated at 26 °C on a shaker at 200 rpm for an additional 48 h. After incubation, levan in groups B to D was extracted as described above and used to determine the change in the distribution of molecular weights.

### Enzyme involved in Levan synthesis and degradation secreted by *L*.*citreum* BD1707

To reduce the interference of levan on the precipitation of enzymes secreted by *L*. *citreum* BD1707 in the broth by ammonium sulfate, the resuspended BD1707 cells were inoculated into tomato juice supplemented with 2% (v/w) sucrose and cultivated at 26 °C for 72 h. The cultivated broth was centrifuged at 15,000×g for 15 min at 4 °C to remove bacterial cells. The proteins in the supernatant was precipitated by ammonium sulfate at 60% saturation and collected by centrifugation at 20,000×g for 30 min at 4 °C. The pellet was dissolved in sodium acetate buffer (20 mM, pH 5.6) containing 2 mM CaCl_2_ and dialyzed (MWCO 14,000 Da) against the same buffer at 4 °C overnight with 2 changes of buffer to remove the residual ammonium sulfate.

The lyophilized protein was dissolved with 1x Laemmli Sample Buffer (Bio-Rad, USA) and incubated at 37 °C for 1 h. For SDS-PAGE, 6% acrylamide containing 0.1% SDS was used. Proteins in the gel were stained with Coomassie blue G-250 and de-stained with 10% acetic acid solution. The standard protein markers were purchased from Bio-Rad (USA).

For in situ detection of the levansucrase activity, the SDS-PAGE gel was incubated in 20 mM sodium acetate buffer containing 50 g/L sucrose buffered at pH 5.6, as described by Dols, Remaud-Simeon, Willemot, Vignon, and Monsan [21]. Briefly, the gel was washed three times with 20 mM sodium acetate buffer (pH 5.6) containing 2 mM CaCl_2_ and 0.1% (vol/vol) Triton X − 100 at room temperature to eliminate the SDS and then soaked in 20 mM sodium acetate buffer (pH 5.6) containing 2 mM CaCl_2_ and 50 g/L of sucrose at 30 °C for 48 h. The protein bands capable of EPS synthesis were detected by the appearance of opaque polymers in the gel [22]. The polysaccharide formed on the gel was extracted by warm water, and structure characterized as described in previous study [19]. The band in a parallel SDS-PAGE gel with corresponding polymerization activity in situ was cut off and processed with peptide mass fingerprinting to determine the sequence of the enzyme [23].

For levan-degrading activity assay, single protein band in the SDS-PAGE gel was cut and individually incubated in one milliliter of 20 mM sodium acetate buffer containing 10 g/L levan (prepared previously) and 2 mM CaCl_2_ (pH 5.6) at 30 °C for 72 h. The released fructose was determined by HPLC method described below [24].

### Analytical methods

The levels of sucrose, glucose and fructose in the cultivated TSJM were determined by a high-performance liquid chromatography (HPLC)-based method described previously [24].

The Mw of the levan obtained was determined by high-performance size exclusion chromatography (HPSEC) using a Perkin-Elmer series 200 liquid chromatography (PerkinElmer, Waltham, MA) equipped with a Perkin-Elmer series 200 refractive index detector. Two TSK-GEL columns (G6000PWXL and G4000PWXL, Tosoh Bioscience Co., Japan) were maintained in series, utilizing 0.1 M NaNO_3_ as the eluent at a flow rate of 0.6 ml/min. The columns were maintained at 25 °C, and 5 mg/ml of BD1707 levan dissolved in the 0.1 M NaNO_3_ was filtered through a 0.22-μm filter before injection. Commercial pullulans with molecular mass ranging from 6000 to 2,560,000 Da (6000 Da, 12,000 Da, 110,000 Da, 800,000 Da, 2,560,000 Da) (Sigma, St. Louis, MO, USA) were used as standards (see Supplementary material, Table S2).

The viable cell counts of BD1707 in the cultivation broth were enumerated by plating the serially 10-fold diluted sample on M17 agar (Merck, USA) and incubating at 30 °C aerobically. The pH was measured by using a pH meter (PB − 10, Sartorius AG, Goettingen, GER) [19].

### Statistical analysis

All experiments and analyses at every time point for each experiment were performed in triplicate. The means, standard errors, and standard deviations were calculated from replicate experiments and analyzed using Design Expert 8.0.5b and OriginPro8.0.

## Results

### Effect of cultivation period, temperature, initial pH and sucrose concentration of TJSM on Levan production

**Fig. 1:**
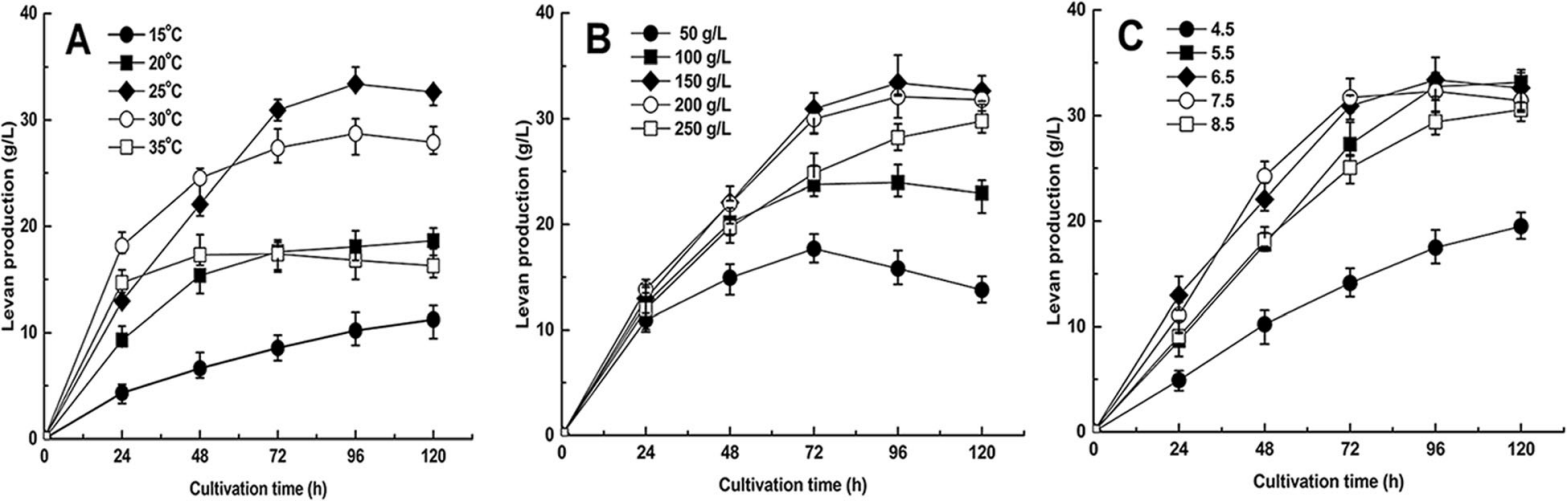
Effects of individual cultivation factors on levan production by BD1707. **a** Cultivation temperature, **b** sucrose concentration and **c** initial pH

Figure 1a illustrated the effect of cultivation temperatures from 15 °C to 35 °C on levan production by the strain *L*.*citreum* BD1707. In TJSM with sucrose at 150 g/L and an initial pH of 6.5, high yields of levan (exceeding 28 g/L) was produced by the strain *L*. *citreum* BD1707 at 30 °C and 25 °C after 96 h of cultivation, while low yields of levan (below 20 g/L) were observed at 15, 20 and 35 °C. A maximal yield of levan (33.40 g/L) was observed at 25 °C, which was chosen as the center point with 5 °C step changes in subsequent tests.

Levan production by the strain *L*.*citreum* BD1707 in TJSM at pH 6.5 with different levels of sucrose (50, 100, 150, 200 or 250 g/L) at 25 °C for 120 h is shown in Fig. 1b. Overall, high levels of sucrose are beneficial for levan synthesis in TJSM by the strain *L*.*citreum* BD1707. The levan yield at 96 h was enhanced from 15.8 to 33.4 g/L by increasing the sucrose concentration from 50 to 150 g/L. Therefore, sucrose concentration of 150 g/L was selected as the center point with a step change of 50 g/L.

The effect of initial pH value (4.5–8.5) on levan production by BD1707 was examined in TJSM with 150 g/L sucrose at 25 °C (Fig. 1c). The highest levan yield (33.40 g/L) was observed in TJSM with an initial pH of 6.5. Therefore, the center point of the initial pH was fixed at 6.5 with step changes set at 1.0.

In the single-factor experiments (Fig. 1a, b, c), high levan yields were observed in all batches after 72–120 h of cultivation, and A peak yield of levan (33.40 g/L) occurred at 96 h. Therefore, 96 h was chosen as the center point of cultivation time with step changes set at 24 h.

### Response surface methodology (RSM)

A three-level four-factor Box-Behnken experimental design for RSM with 27 runs was employed to determine the optimal cultivation variables for levan production by *L. citreum* BD1707 in TJSM. The results derived from the experimental data and simulated values predicted by the constructed model employing levan yield as the response variable were listed in Table 3. By applying multiple regression analysis on the experimental data, a second-order polynomial equation describing the relationship of levan production (Y) in TJSM with cultivation time (A), cultivation temperature (B), initial pH (C) and sucrose concentration (D) is established in terms of coded factors as eq. 2.

2 $$ \mathrm{Y}=33.36+0.68\mathrm{A}+4.95\mathrm{B}+0.16\mathrm{C}+3.92\mathrm{D}-0.13\mathrm{AB}-1.54\mathrm{AC}+0.66\mathrm{AD}-0.29\mathrm{BC}+0.18\mathrm{BD}-0.28\mathrm{CD}-1.13{\mathrm{A}}^2-9.26{\mathrm{B}}^2-1.42{\mathrm{C}}^2-5.11{\mathrm{D}}^2 $$

As shown in Table 4, based on the analysis of *p*-value and *F*-value, the four factors were ranked in order of positive effect on levan production as follow: B (Cultivation temperature, *F*-value of 299.24, *p*-value<0.0001)>D (sucrose concentration, *F*-value of 187.95, *p*-value<0.0001)>A (cultivation time, *F*-value of 5.71, *p*-value of 0.0342)>C (initial pH value, *F*-value of 0.3, *p*-value of 0.594). Linear effect of cultivation temperature and sucrose concentration was highly remarkable, indicating that they might act as limiting factors on levan yield. According to the coefficients of interactions, AD (0.66) and BD (0.18) had positive effect on levan yield, while negative effect could be seen in the AB (− 0.13), AC (− 1.54), BC (− 0.29) and CD (− 0.28), but all the interactions had no significant effect on responses.

**Table 4:** Model coefficient estimated by multiple linear regression

Factor	Coefficient estimate	Standard error	F value	*p*-value
Intercept	33.36	0.57	–	–
A	0.68	0.29	5.71	0.0342
B	4.95	0.29	299.24	<0.0001
C	0.16	0.29	0.3	0.594
D	3.92	0.29	187.95	<0.0001
AB	−0.13	0.50	0.069	0.7975
AC	−1.54	0.50	9.66	0.0091
AD	0.66	0.50	1.77	0.2076
BC	−0.29	0.50	0.34	0.5692
BD	0.18	0.50	0.13	0.7227
CD	−0.28	0.50	0.32	0.5824
A^2^	−1.13	0.43	6.98	0.0215
B^2^	−9.26	0.43	465.31	<0.0001
C^2^	−1.42	0.43	11	0.0061
D^2^	−5.11	0.43	141.87	<0.0001

By means of analysis of variance (ANOVA), the quadratic regression model with a low p-value (*p* < 0.0001) and insignificant result from the lack-of-fit test (*p* = 0.1295) was proven to be suitable and had good prediction ability. The coefficient of determination (R^2^) measuring the model’s goodness of fit was 0.9887, which indicated that the model was capable of explaining 98.87% of the variation in the response and that only 1.13% of the total variations could not be accounted for by the model. The “adjusted R^2^” and the “predicted R^2^” were 0.9756 and 0.9363, respectively, which indicated that the model was highly reliable according to the principle of “the nearer to 1.0 the R^2^ value was, the more fit the model was deemed to be”. The “adequate precision” value of the present model, reflecting the signal-to-noise ratio, was 31.217, which was much greater than the desirable value of 4, suggesting that the model could be used to navigate the design space. The standard deviation, mean, and predicted residual sum of squares (PRESS) values were 0.99, 25.84, and 66.74, respectively, and the low variation coefficient value (C.V. =3.84) provided further evidence for the high preciseness and reliability of the model.

**Fig. 2:**
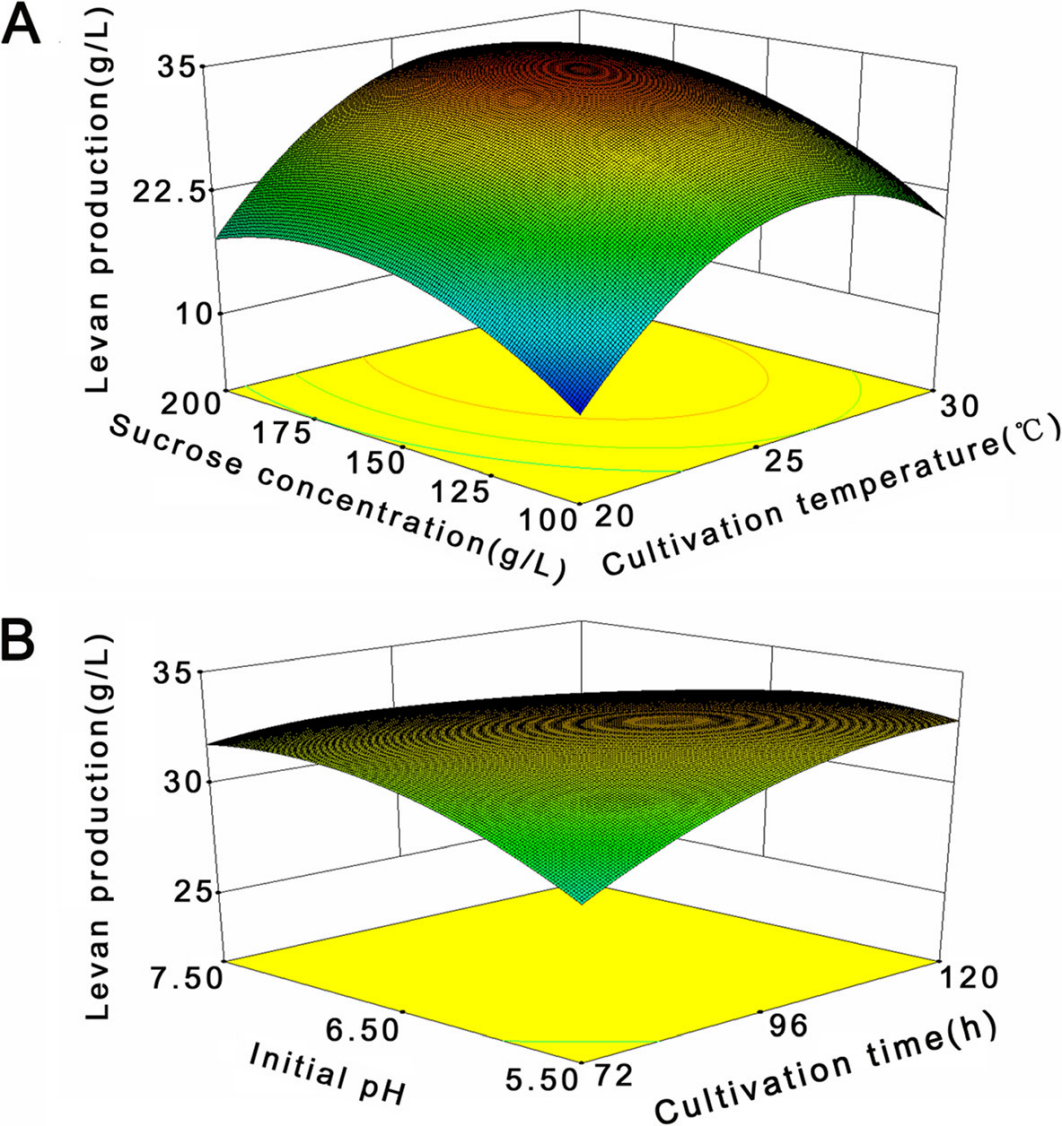
3D response surface plots of the combined effects of independent factors on levan production. **a** Sucrose concentration and cultivation temperature, **b** initial pH value of the medium and cultivation period

Six response surface graphs were obtained from this model, two of which were chosen to illustrate the combined effects of individual variables on levan production. Figure 2a shows the effect of sucrose concentration and cultivation temperature on the response at the fixed center values of initial pH and cultivation time. The yield of levan was correlated with increasing sucrose concentrations and cultivation temperatures up to approximately 150 g/L and 25 °C, respectively, while the levan yields decreased with further increase in the levels of these two variables. The interaction effect of initial pH value and cultivation time on levan production was also explored while keeping the sucrose concentration and cultivation temperature constant at the center values (Fig. 2b). Increasing the initial pH value and cultivation time led to an increase in levan production but at a modest rate compared with the increase in sucrose concentration and cultivation temperature. A maximum levan yield of 35.10 g/L by the strain *L*.*citreum* BD1707 was predicted by the point prediction tool of Design Expert software under the optimized cultivation conditions, i.e., cultivation time of 112 h, cultivation temperature of 26 °C, initial pH value of 6.12 and sucrose level of 172 g/L.

### Model validation

**Fig. 3:**
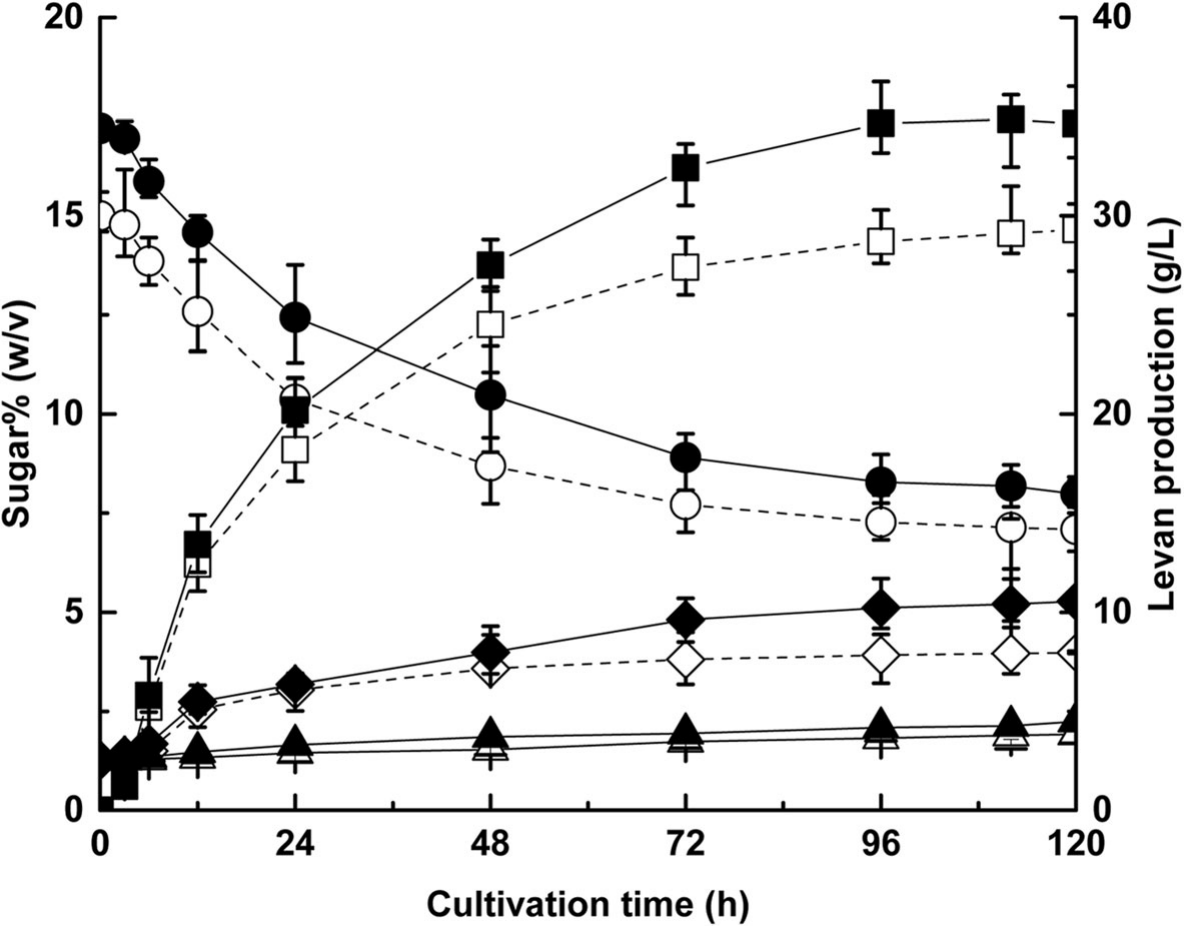
Changes in sucrose, glucose, fructose and levan production by *L. citreum* BD1707 under optimal and nonoptimal cultivation conditions (——, optimal; ———, nonoptimal; ■□, levan production; ●○, sucrose; ◆◇, glucose; ▲△, fructose)

To validate the second-order model, three independent replications were conducted under optimal conditions. Meanwhile, cultivation in triplicate under the non-optimized conditions described previously [19] was also carried out as a control. Figure 3 provides a comparison of the changes in the levels of sucrose, glucose and levan between optimized and non-optimized fermentation.

The highest yield of levan from the strain *L*.*citreum* BD1707 under optimized cultivation conditions was 34.86 g/L, approximately the maximum value (35.10 g/L) predicted by the second-order model, which was reached at a cultivation time of 112 h and was much higher (*P* < 0.05) than the yield obtained under non-optimized conditions.

Meanwhile, the sucrose concentration decreased by 9.22% (w/v) from the initial level of 17.20% (w/v) to 7.98% (w/v) at 120 h under the optimized conditions, while only a decrease of 6.92% (w/v) in sucrose concentration was observed in non-optimized cultivation conditions, which could be resulted from the high expression and/or high enzyme activity of levansucrase in BD1707 under optimal conditions. Levansucrase, a member of the glycoside hydrolase family 68, is crucial in the formation of levan with two catalytic functions: hydrolysis of sucrose and transglycosylation of the fructose moiety of sucrose to the elongated fructan chain. This result indicated that process optimization is an efficient way to increase levan production. Additionally, glucose, one of the hydrolysis products of levansucrase, accumulated gradually during cultivation, and the increment of 4.03% (w/v) under optimal conditions was markedly higher (*p* < 0.05) than that (2.71% (w/v)) under non-optimized conditions, which provided further evidence that the activity of levansucrase expressed by the strain *L*.*citreum* BD1707 is stronger under optimized conditions than that under non-optimized conditions. Unlike glucose, no significant change in the concentration of fructose was observed under these two cultivation conditions, which fluctuated around 1% (w/v).

### Degradation of the Levan during prolonged cultivation of the strain *L*.*citreum* BD1707

**Fig. 4:**
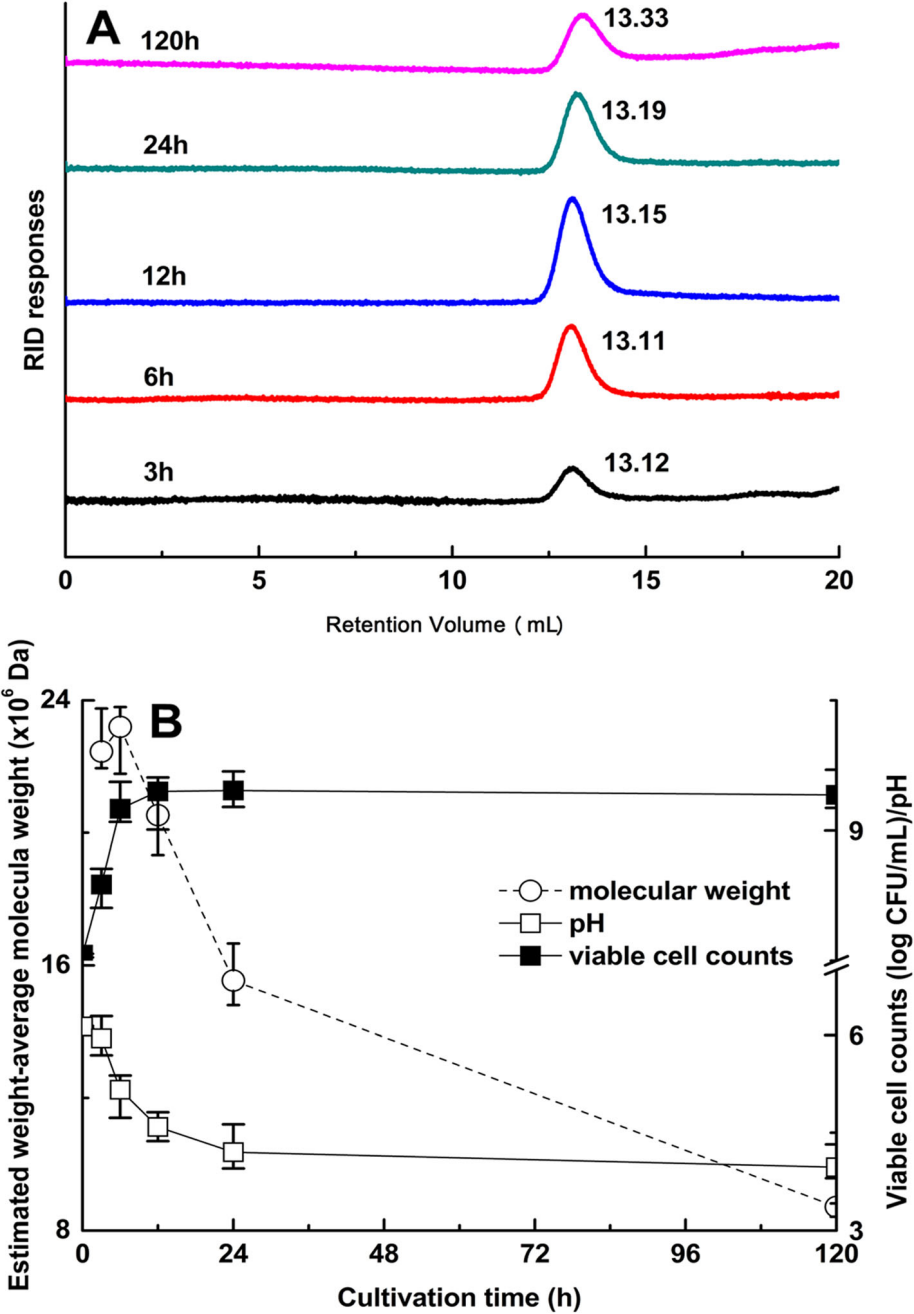
Changes in the Mw of BD1707 levan, pH and viable cell counts during fermentation under optimal conditions. **a** Retention volume of levan in different cultivation periods; **b** calculated Mw, pH and viable cell counts

To investigate the changes in Mw of levan synthesized under optimal conditions, BD1707 was cultivated in TJSM (pH 6.12) containing 172 g/L sucrose at 26 °C and 200 rpm for 120 h. Samples at different intervals (3, 6, 12, 24 and 120 h) were obtained, and the molecular weight distribution of levan was analyzed. As shown in Fig. 4, the calculated Mw of levan obtained at different cultivation periods were 2.245 × 10^7^ Da (3 h), 2.320 × 10^7^ Da (6 h), 2.053 × 10^7^ Da (12 h), 1.554 × 10^7^ Da (24 h) and 8.809 × 10^6^ Da (120 h) , indicating the molecular mass of levan synthesized by the strain *L*.*citrem* BD1707 reached the highest value at 6 h of cultivation and then decreased with prolonged cultivation.

### Influence of Levan-degrading enzymes and organic acids on the mw of Levan

To explore the factor responsible for the degradation of levan, cultivated TJSM by the strain *L*.*citreum* BD1707 at 72 h was chosen for further investigation. At this stage, cells of the strain *L*.*citreum* BD1707 were in the declining phase (shown in Fig. 4b), inclining to secret some degrading enzymes including the presumed levan-degrading enzymes (levansucrase and/or levanase, induced by sucrose and/or levan, respectively) to salvage the residual nutrients in the environment to maintain the viability of the bacterial cells. On the other hand, at this stage, the broth was with a rather low pH value of approximately 4.0, an ideal acidic environment to explore the impact of organic acid on the change of the Mw of existed levan in the broth during elongated cultivation.

**Fig. 5:**
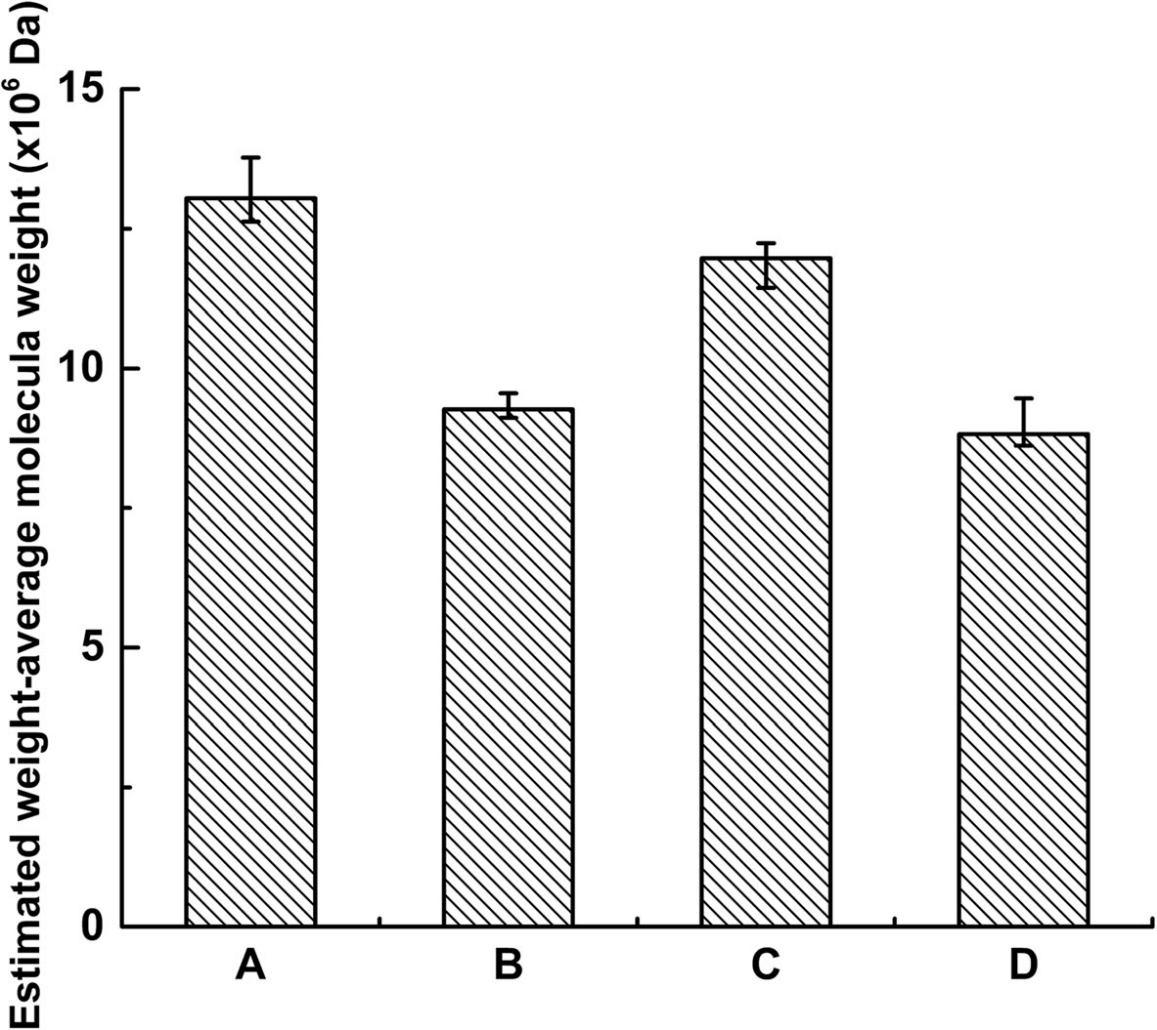
Molecular weight distribution of levan with different treatments. **a** Levan from 72-h cultivation sample; **b** 72-h cultivation sample centrifuged, filtered through a 0.22-μm membrane, and incubated for 120 h; **c** 72-h cultivation sample centrifuged, filtered through a 0.22-μm membrane, pH-adjusted to 7.0, boiled, pH-adjusted to 4.0, and incubated for 120 h; **d** control

As shown in Fig. 5, the Mw of levan decreased markedly from 1.305 × 10^7^ Da at 72 h of cultivation (levan from group A) to 8.824 × 10^6^ Da at 120 h of cultivation (levan from group D), which might be attributed to the synergetic hydrolysis by both secreted enzymes (levanase/levansucrase) and organic acids. The Mw of levan from group C from the filter sterilized, enzyme inactivated and pH-readjusted medium was 1.197 × 10^7^ Da, which was slightly lower than that of levan from group A, indicating that organic acids were not responsible for the degradation. Compared with the levan from group A, a significantly higher degree of levan degradation than that from group B could be observed (9.268 × 10^6^ Da, obtained in the existence of pH value of 4.0 and degrading enzyme for an additional 48 h). As organic acid existed in both Group B and Group C, except for the presumed degrading enzyme(s) which was heat inactivated in the latter, the Mw of the levan in group B was much lower than that of levan in group C strongly suggested that the existence of levan degrading enzyme(s) (levanase/levansucrase) secreted into the medium during the first 72 h of cultivation. This result was further supported by the fact that the Mw of levan in group B was almost consistent with that in sample D.

### Enzymes involved in Levan synthesis and degradation by *L*.*citreum* BD1707

**Fig. 6:**
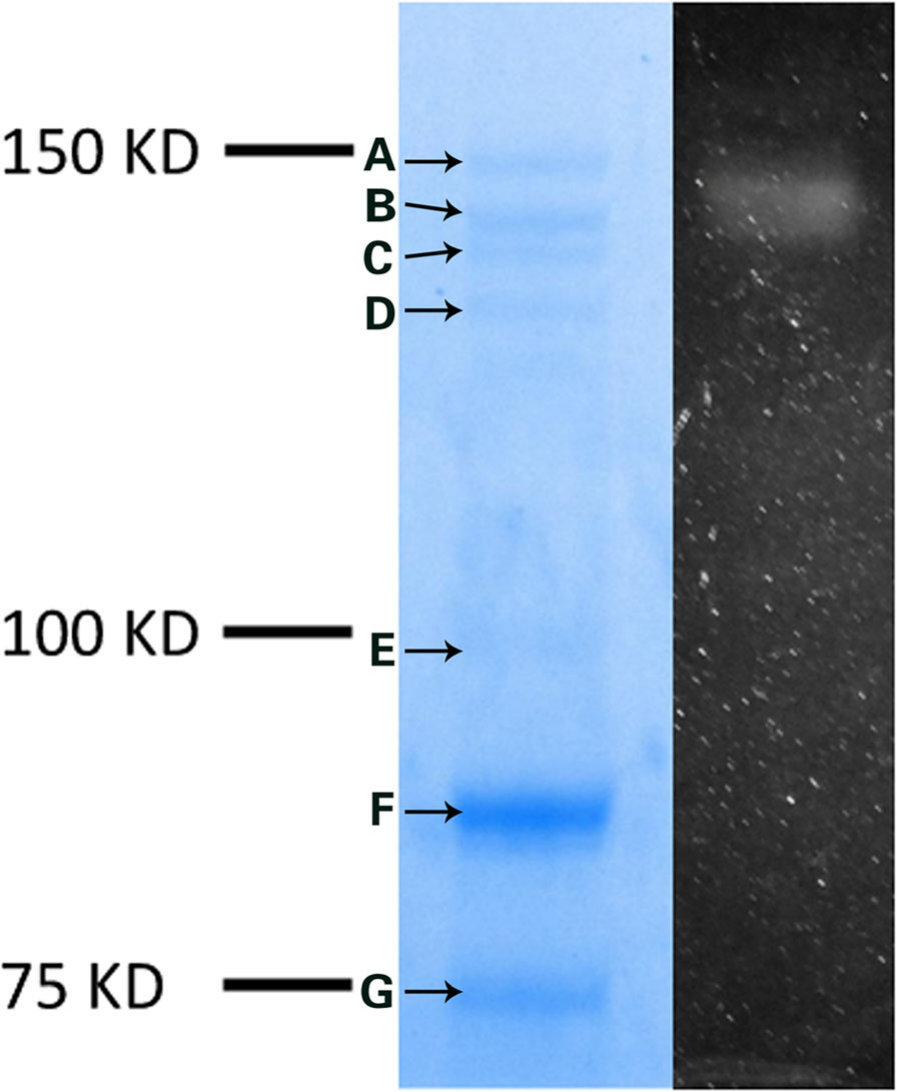
In situ gel polysaccharides synthesis

The protein expressed by *L*.*citreum* BD1707 in tomato juice supplemented with 2% (w/v) sucrose could be more efficiently precipitated by ammonium sulfate at 60% saturation (see supplementary material, Fig. S3). As shown in Fig. 6, although seven visible protein bands (Band A to G) were observed on the stained SDS-PAGE gel, only one obvious white and opaque band occurred at the position of 130 KD in the unstained SDS-PAGE gel soaked in a 50 g/L sucrose solution for 48 h at 30 °C. This result indicated the protein band at this position possessed polymerization activity. The polymers in the gel were extracted and characterized as levan (data not shown). After cutting off and subjecting to peptide mass fingerprinting, the corresponding SDS-PAGE protein band (Band B) was identified as levansucrase (tr|A0A192S224|A0A192S224_LEUME) of *L.mesenteroides* with a sequence coverage of 25%, which indicated the gene encoding the levansucrase in *L*. *citreum* BD1707 is phylogenetically diversified from its alleles in other levan producing bacteria. The levansucrase showed an unusual Mw of 130 KD, which was greatly differed from the reported Mw of levansucrase, usually in the range of 50-90KD, secreted by other levan producing bacterial species [25–27]. The individual protein band (Band A to G) on the stained SDS-PAGE gel was cut off and assayed for their fructose releasing activity from levan. Among the seven protein bands, only band B showed detectable levan-degrading activity, which released about 0.04% (w/v) fructose from levan during the incubation of the band with 10 g/L levan at 30 °C for 48 h.

## Discussion

To enhance the levan yield by the strain *L*. *citreum* BD1707 in tomato juice, 4 cultivation factors i.e. cultivation period, cultivation temperature, initial pH and sucrose concentration were selected from a variety of nutrition and cultivation parameters to investigate their impacts on the synthesis of levan. In the single factor test, cultivation temperature and the level of sucrose exert more significant impact on the levan yield of the strain L.citreum BD1707. In the tested cultivation temperature of 15-35 °C, 25-30 °C is more suitable for *L*. *citreum* BD1707 to synthesize levan in the presence of sucrose. As temperature is one of the most important environmental factors affecting the growth and development of bacterial cells by influencing intracellular bioactivities [28], alike other *Leuconostoc* species, *L. citreum* is a mesophilic LAB, preferring to grow, proliferate and accumulate secondary metabolites such as dextran [29], inulin [30], mannitol [31] and bacteriocin [32] at mild temperatures. The low yield of levan of *L*. *citreum* BD1707 at cultivation temperature outside this scope might be due to 1) decreased levansucrase expression caused by slow bacterial growth at low temperature [33], 2) inactivation of extracellular levansucrase at elevated temperatures, 3) and irreversible denaturation of levansucrase to synthesize levan at higher cultivation temperature [34].

Sucrose was determined to be the sole carbon source for the strain *L*.*citreum* BD1707 to synthesize levan in our previous study [19], which was in consistent with the results of other researchers [2]. Consequently, sucrose was selected as a crucial variable to optimize levan production in this study. The low yield of levan by the strain *L*.*citreum* BD1707 at sucrose levels lower than 150 g/L might be attributed either to the insufficient substrates for the synthesis of the fructan-type polymer or the rapid hydrolysis of the synthesized polymer by enzymes with levan degrading activity, i.e. levanase [35] and levansucrase [36] during cultivation. In our study, the degradation of the synthesized levan seemed less obvious in TJSM with a high concentration of sucrose (≥ 150 g/L), which might be caused by the repressed hydrolytic activity of the enzyme at the presence of high levels of reducing sugar generated during the cultivation process [37] (Fig. 1b). A further increase in sucrose concentration (250 g/L) would result in a high osmotic pressure unsuitable to the growth and metabolism of the strain *L*.*citreum* BD1707, and thus led to a lower yield of levan after 96 h of cultivation, as also observed in cultivation of other microbes such as *Zymomonas spp*. in the presence of high osmotic pressure [38, 39]. A similar result was obtained by SHIH et al., who observed that the yield of levan by *Bacillus subtilis* (natto) decreased at both extremely high and low sucrose concentrations [40].

The initial pH value of the culture medium might also affect the yield and size of the soluble levan synthesized by microorganisms [41] by altering the morphologic features of the producer cells [39]. At all tested pH values, levan production was higher than 29 g/L after 96 h except in TJSM with an initial pH of 4.5 (17.48 g/L), which might be due to either poor growth of BD1707 cells and /or the levan polymerization activity of levansucrase being lower than the hydrolytic activity [37]. Nevertheless, the levan yield by the strain *L*.*citreum* BD1707 at pH 4.5 was still higher than those of other microbial producers [37]. A plausible explanation to this phenomenon might be the strong acidity tolerance ability of *L. citreum*, which endows the bacteria in this species to grow well in low-pH niche and subsequently inhibit the growth of other spoilage microbes and thus prolong the shelf life of kimchi [42]. The fluctuation in levan production by the strain *L*.*citreum* BD1707 in TJSM with initial pH values of 5.5 to 8.5 during the cultivation exhibited a similar tendency, with levan yield peaking at 96 h and decreasing thereafter, indicating that the strain *L*.*citreum* BD1707 could synthesize levan in a wide range of pH values.

Levan degradation might be related to two biochemical process: (1) hydrolysis by levan-degrading enzymes secreted by BD1707, alike those expressed by other levan producers via a carbohydrate regulation mechanism [35, 36]; (2) acid hydrolysis by organic acids [15]. In the present work, while the Mw of levan peaked at 6 h of cultivation (6 h), the strain *L*.*citreum* BD1707 underwent dramatically rapid growth (*P* < 0.05), with the viable cell counts increasing from an initial value of 7.6 log10 to 9.25 log10 cfu/mL. At this point of cultivation, the growth of the strain *L*.*citreum* BD1707 was at the late exponential phase (Fig. 4b), with a levan yield of 5.8 g/L (Fig. 3), indicating the existence of levansucrase in the cultivation medium. Meanwhile, the pH of the medium decreased sharply from 6.13 to 5.16, which might be low enough to shift the catalytic activity partially from polymerization to degradation upon further cultivation [15].

As the Mw of levan of 1.305 × 10^7^ Da synthesized by the strain *L*.*citreum* BD1707 at 72 h (levan from group A) was decreased to 8.824 × 10^6^ Da at 120 h of cultivation (levan from group D) in the presence of both organic acids and continued secretion of presumed levan degrading enzyme, whilst the Mw of the levan sample shifted slightly to 1.197 × 10^7^ Da (levan from group C) in the absence of the presumed levan degrading enzyme, compared to a sharply decrease of Mw to 9.268 × 10^6^ Da in levan levan from group B in the presence of presumed degrading enzyme(s) at the same acidic environment. Therefore, the degradation of the existed levan could in reasonably linked to the activity of some enzyme secreted into the broth by the levan producer. Our result was in disagreement with those of Bekers et al. and Runyon et al., who concluded that acid hydrolysis was the main levan-degrading factor [43] and that levan degradation was more likely to occur at pH values lower than 5.5 and faster at even lower pH values [15]. The discrepancy of our result and the published literature can be plausibly explained by the fact that *L*. *citreum* is acid tolerant while producers such as *Zymomonas mobilis* produced little organic acid into its growth medium [43].

By means of SDS-PAGE and in situ polymerization, an active protein band responsible for levan synthesis was observed in the cultivated TJSM with 20 g/L sucrose by at 30 °C for 72 h by the strain *L*. *citreum* BD1707 and identified as levansucrase, which also showed detectable fructose releasing activity from levan. The identified levansucrase was with an unusual Mw of 130 KD, much higher than the reported Mw of levansucrase secreted by other levan producing bacterial species [25–27], which was usually in the range of 50–90 KD and also with low amino acid sequence coverage with that reported in *L*. *mesenteroides*. Our result is in agreement with previously published literature in that levansucrase could display hydrolytic activity just as that of levanase. Mendez-Lorenzo L. et al. found that while SacB (*Bacillus subtilis* 168 levansucrase) released 1 μmol of fructose per min from 100 mg/mL of sucrose, the enzyme released 1/18 μmol of fructose from 100 mg/mL of levan. The authors hypothesized that the levan hydrolytic activity of levansucrase was minor at the first stage of reaction with enough sucrose as substrate for the polymerization, but it might become obvious with the decrease of sucrose and the increase of levan in the middle or late stages of the reaction [44]. In blasting the full genome sequence of *L*. *citreum* BD1707, a fragment of DNA in the genome was inferred to encode a protein related to levanase with protein ID of WP_040177126.1. Unfortunately, in our study, no levanase was precipitated from the tomato juice with 20 g/L sucrose cultivated with *L*. *citreum* BD1707 for 72 h. A plausible explanation might be levan synthesized by *L*. *citreum* BD1707 at the presence of 20 g/L of sucrose is insufficient to induce the bacteria cells to express detectable levanase.

The functions of exopolysaccharides, especially those homopolymer of EPS were molecular weight depended. Generally, levan with high molecular weight was commonly used as encapsulating agent, emulsifier, stabilizer and thickener for its specific rheological and physical-chemical properties [45], whilst levan with relatively low molecular weight behaved more efficiently in healthy promotion, which is usually employed as dietary fiber or prebiotics with extremely lower degree of polymerization. Esawy M.A. et al. proved that levan (9.53 KD) from *Bacillus subtilis* M was promising inhibitor of cytochrome for its inhibitory effect on carcinogens induced-DNA fragmentation [46]. The antitumor activity of levan (456,900 Da) was much stronger than that of other levan samples with higher molecular weights (720,200 Da, 769,500 Da and 1,073,500 Da) [17]. Levan of 2.25 × 10^6^ Da molecular weight exhibited a moisturizing effect as well as a similar cell proliferation effect on human fibroblast and keratinocyte cells [6]. Therefore, factors affecting the Mw of levan in the large scale preparation of this polymer should be noticed as molecular weight might influence its bioactivity and functionality.

## Conclusion

TJSM was previously proven to be a low-cost medium suitable for *L. citreum* BD1707 growth for levan synthesis. In the present study, RSM based on a 27-factorial BBD was successfully employed to optimize the cultivation conditions to further enhance levan production by BD1707. The optimal cultivation conditions were predicted to be as follows: cultivation time, 112 h; cultivation temperature, 26 °C; initial pH, 6.12; and sucrose concentration, 172 g/L. Under the optimized cultivation conditions, a maximum levan yield of 34.86 g/L was attained. During the cultivation of BD1707 in TJSM, the Mw of the levan produced by BD1707 reached a maximum value of 2.320 × 10^7^ Da at 6 h of cultivation and then gradually decreased to 8.809 × 10^6^ Da at 120 h of cultivation. Levansucrase with Mw of 130 KD secreted by the strain L.citreum BD1707 into the medium during cultivation was presumed for the degradation of levan, leading the levan with a lower molecular weight, while the hydrolysis at low pH caused by organic acid accumulation could be neglected.

## Supplementary Information

**Additional file 1: Table S1.** Major Biochemical components and parameters of tomato juices prepared from different variety of *Lycopersicon esculentum* (values are the average ± range of triplicate analyses). **Figure S1**. Different variety of *Lycopersicon esculentum* and the prepared tomato juice (I: *Lycopersicon esculentum var. vulgare,* II: *Lycopersicon esculentum var. grandifolium,* III:*Lycopersicon esculentum var. valiudmbaily*). **Figure S2.** GPC-HPLC profiles of pullulan with molecular mass ranging from 6,000 to 2,560,000 Da. **Figure S3.** SDS-PAGE profiles of proteins expressed by the strain *L*.*citreum* BD1707 in juices supplemented with 2% (w/v) sucrose. The proteins were precipitated from the supernatant of the cultivated TJSM either by ammonium sulfate at 40% (40%ASP) or 60% saturation (60%ASP).

## Data Availability

The 16sRNA sequence of *L*. *citreum* BD1707 has been deposited at DDBJ/ENA/GenBank under the accession KT626384.The whole genome information of *L*. *citreum* BD1707 has been deposited at DDBJ/ENA/GenBank under the accession JACDIP000000000. All supporting data are included in the manuscript and supplementary materials.

## Abbreviations

ANOVA: Analysis of variance
BBD: Box-Behnken design
CDM: Chemically defined medium
CGMCC: China general microorganism culture collection
EPS: Exopolysaccharide
FOS: Fructooligosaccharide
GH32: Glycoside hydrolase family 32
GH68: Glycoside hydrolase family 68
KD: Kilo Dalton
LAB: Lactic acid bacteria
Mw: Molecular weight
PRESS: Predicted residual sum of squares
RSM: Response surface methodology
SDS-PAGE: Sodium dodecyl sulfate polyacrylamide gel electrophoresis
TJSM: Tomato juice-sucrose medium
