# Supplementary material for: Levan from Leuconostoc citreum BD1707: production optimization and changes in molecular weight distribution during cultivation
Source: BMC Biotechnol. 2021 Feb 4;21:14. doi: 10.1186/s12896-021-00673-y (PMC7863327; doi:10.1186/s12896-021-00673-y)
Supplement: Supplementary file 1 — Additional file 1: Table S1. Major Biochemical components and parameters of tomato juices prepared from different variety of Lycopersicon esculentum (values are the average ± range of triplicate analyses). Figure S1. Different variety of Lycopersicon esculentum and the prepared tomato juice (I: Lycopersicon esculentum var. vulgare, II: Lycopersicon esculentum var. grandifolium, III:Lycopersicon esculentum var. valiudmbaily). Figure S2. GPC-HPLC profiles of pullulan with molecular mass ranging from 6,000 to 2,560,000 Da. Figure S3. SDS-PAGE profiles of proteins expressed by the strain L.citreum BD1707 in juices supplemented with 2% (w/v) sucrose. The proteins were precipitated from the supernatant of the cultivated TJSM either by ammonium sulfate at 40% (40%ASP) or 60% saturation (60%ASP). [file 12896_2021_673_MOESM1_ESM.docx]

Table S1 Major Biochemical components and parameters of tomato juices prepared from different variety of *Lycopersicon esculentum* (values are the average ± range of triplicate analyses).

Figure S1 Different variety of *Lycopersicon esculentum* and the prepared tomato juice (Ⅰ: *Lycopersicon esculentum var. vulgare,* Ⅱ: *Lycopersicon esculentum var. grandifolium,* Ⅲ：*Lycopersicon esculentum var. valiudmbaily*)

Figure S2 GPC-HPLC profiles of pullulan with molecular mass ranging from 6,000 to 2,560,000 Da

Figure S3 SDS-PAGE profiles of proteins expressed by the strain *L*.*citreum* BD1707 in juices supplemented with 2% (w/v) sucrose. The proteins were precipitated from the supernatant of the cultivated TJSM either by ammonium sulfate at 40% (40%ASP) or 60% saturation (60%ASP).

Table S1

| Component/parameter | Tomato juice  in our paper | Tomato juice I | Tomato juice II | Tomato juice III |
| --- | --- | --- | --- | --- |
| Density(g/100 mL) | 1.018±0.002 | 1.028±0.004 | 1.008±0.002 | 1.020±0.003 |
| Ash (g/100 mL) | 0.42±0.01 | 0.46±0.02 | 0.38±0.03 | 0.44±0.01 |
| Free reducing sugars  (g/100 mL) | 2.67±0.03 | 2.75±0.06 | 2.37±0.05 | 2.70±0.03 |
| Total Kjeldahl nitrogen  (g/100 mL) | 0.074±0.001 | 0.078±0.002 | 0.073±0.002 | 0.077±0.001 |
| Fat (g/100 mL) | 0.10±0.01 | 0.11±0.01 | 0.08±0.01 | 0.10±0.01 |
| pH | 4.15±0.03 | 4.36±0.03 | 4.24±0.01 | 4.34±0.02 |


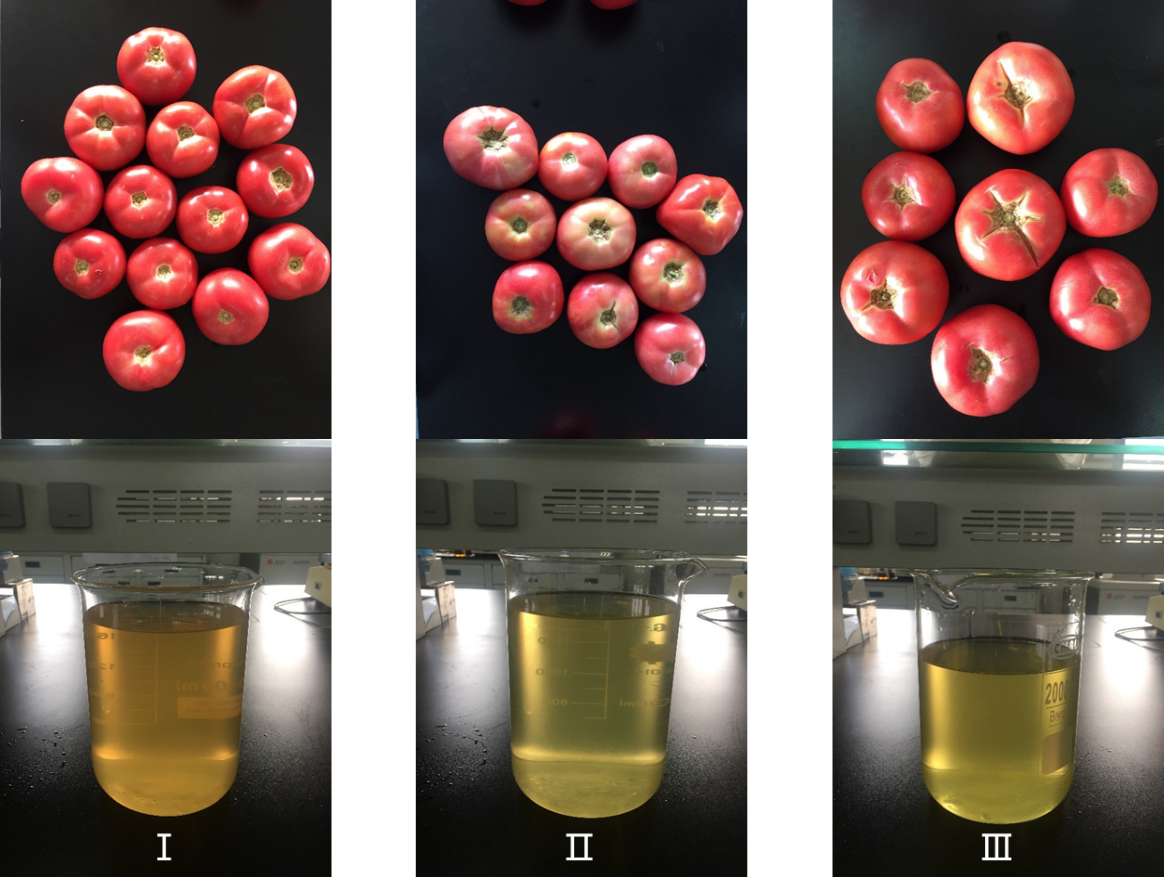


Figure S1


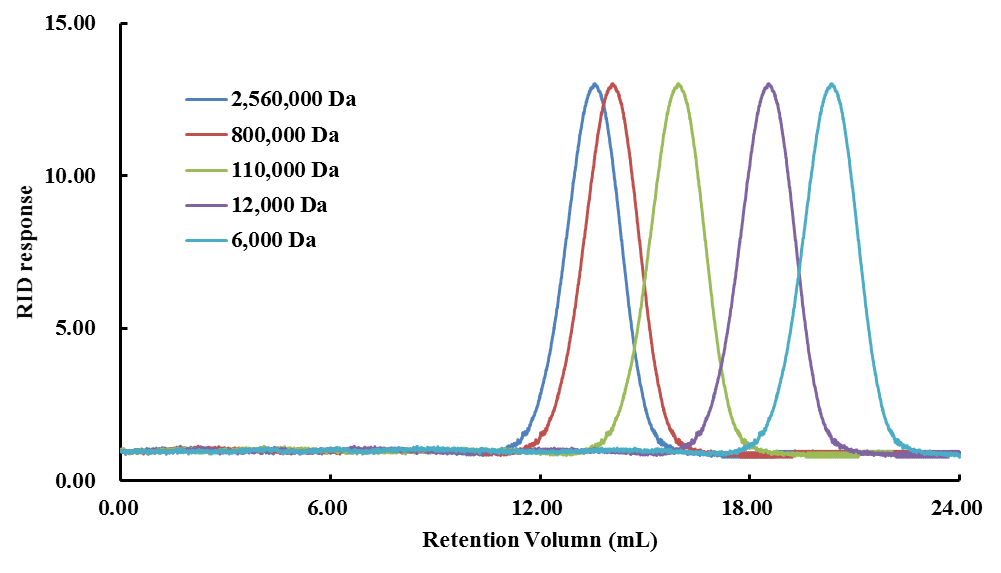


Figure S2


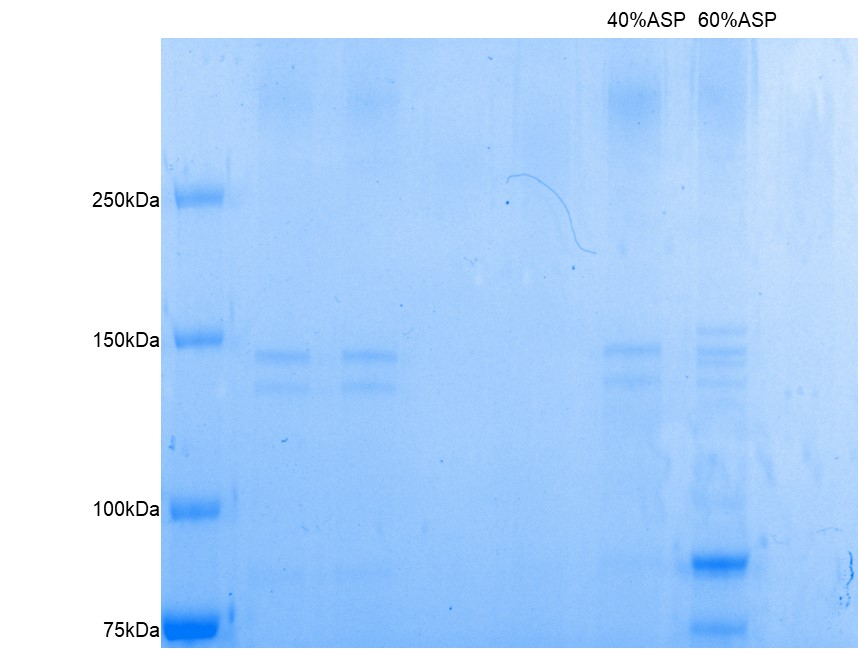


Figure S3
